# Influence of Mechanical Damage under Repeated Loading on the Resistance of Geogrids against Abrasion

**DOI:** 10.3390/ma14133544

**Published:** 2021-06-25

**Authors:** David Miranda Carlos, Filipe Almeida, José Ricardo Carneiro, Maria de Lurdes Lopes

**Affiliations:** Construct-Geo, Department of Civil Engineering, Faculty of Engineering, University of Porto, Rua Dr. Roberto Frias, 4200-465 Porto, Portugal; davidmc@fe.up.pt (D.M.C.); rcarneir@fe.up.pt (J.R.C.); lcosta@fe.up.pt (M.d.L.L.)

**Keywords:** geosynthetics, geogrids, degradation agents, mechanical damage, abrasion, tensile behaviour, reduction factors

## Abstract

Geogrids are building materials widely used for soil reinforcement that can be affected by the action of many degradation agents throughout their service life. The potential negative effect of the degradation agents should be properly estimated and accounted for during the design phase. The main aim of this work was to study the influence of mechanical damage under repeated loading on the resistance of geogrids against abrasion. Three geogrids (one extruded and two woven) were exposed in isolation to mechanical damage under repeated loading and abrasion tests, followed by the successive exposure to both degradation tests. The damage suffered by the geogrids was evaluated by visual inspection and by tensile tests. Based on the changes found in tensile strength, reduction factors were determined. The reduction factors obtained directly from the successive exposure were compared to those resulting from a method in which the reduction factors obtained for the isolated effect of each degradation agent were multiplied. Results indicated that the abrasion process tended to be affected by a previous exposure to mechanical damage under repeated loading and that the multiplication of the reduction factors obtained for the isolated effects of the degradation agents may not correctly represent their combined effect.

## 1. Introduction

Geosynthetics are building materials with a broad use within the context of civil and environmental engineering applications. The aspects associated with their raw materials, manufacturing processes, or the characterization of their properties have been deeply scrutinized in the literature [1,2,3]. Geogrids are a group of geosynthetics that are used for soil reinforcement. Their tensile properties make them suitable materials to improve the mechanical properties of soils, i.e., geogrids can be used to promote an increase in the strength of soils and a decrease in their deformation.

Geogrids might come into contact with several degradation agents throughout their service life, which can produce negative effects on their properties. This potential unpleasant outcome might trigger the instability of the structures in which they are applied. Examples of physical and chemical degradation agents include water and other solvents, temperature, oxygen, and ultraviolet radiation. Yet, there are also mechanical agents that might have negative effects on geogrids, such as the installation on-site process, abrasion, or creep [2,3,4]. As is understandable, geogrids can come into contact with one degradation agent or with several simultaneously. The effect of each degradation agent must be properly estimated and accounted for by means of reduction factors that are used for the design [5,6,7,8]. The technical report ISO/TR 20432 [6] provides the following equation for calculating the long-term tensile strength (*T_D_*) of geosynthetics in the case of being used for soil reinforcement:

(1) $$T_{D}=\frac{T}{{\mathrm{RF}}_{\mathrm{CR}}\times {\mathrm{RF}}_{\mathrm{ID}}\times {\mathrm{RF}}_{W}\times {\mathrm{RF}}_{\mathrm{CH}}\times f_{s}},$$

where T is the characteristic tensile strength and *f_s_* is a factor of safety to allow for extrapolation uncertainty associated with creep rupture and accelerated chemical data. RF_CR_, RF_ID_, RF_W_, and RF_CH_ are reduction factors taking into account the effect of, respectively, creep, mechanical damage, weathering, and chemical and biological agents. Equation (1) shows that the combined effect of different degradation agents is considered by multiplying the reduction factors associated with each one of those agents (each reduction factor represents the isolated effect of the respective degradation agent). Despite being a common method, it might not be the most accurate for taking into account the combined effect of two, or more, agents (because of the interactions that might occur between them). Indeed, the conclusions of previous investigations have shown that this method for obtaining the reduction factors accounting for the combined effect of different degradation agents might either be conservative [9,10,11] or lead to an underestimation [12,13,14].

Abrasion is a degradation process that, in some applications, can occur over the entire service life of geogrids. For instance, the movement of cars or trains may result in the application of cyclic loads over the reinforced infrastructures in which they circulate, fostering the development of abrasion on geogrids. The process of installation on-site of geogrids, including their handling and application, and the placement and compaction of soils over them (these activities include the use of heavy equipment that may induce damage to the reinforcement materials) can also cause abrasion by means of the mobilization of frictional forces between the geogrids and the soils (the abrasion induced to the geogrids upon installation occurs in a specific time frame). Other types of damage besides abrasion occurring during installation on-site of geogrids include, for instance, cuts in fibres or ribs and breaks in junctions (the junctions are where the machine and cross-machine direction ribs meet and are connected). In applications in which abrasive actions are expected to occur over time, it is important to understand the effect of the installation process on the subsequent resistance of geogrids against abrasion. Considering this need, abrasion resistance tests should be carried out not only on intact samples of geogrids, but also on samples previously submitted to the installation process, which might have already suffered some damage.

Laboratory tests can be used to simulate both the damage induced to geogrids during installation activities as well as abrasion occurring over time. Standard EN ISO 10722 [15] presents a methodology to cause mechanical damage under repeated loading on geosynthetics. Huang [16] and Huang and Chiou [17] used the method displayed in ENV ISO 10722-1 [18] (which was later replaced by EN ISO 10722 [15]) for assessing the damage occurring upon installation. Field installation damage tests have also been carried out on geogrids [19,20,21,22]. Some authors have tried to obtain relationships between the damage induced to the materials during the previously mentioned laboratory tests and field experiences [19,21,23]. On the other hand, Rosete et al. [24], Pinho-Lopes and Lopes [25], and Almeida et al. [13] carried out the procedures included in EN ISO 13427 [26], based on wearing the geogrids by friction against a surface, to simulate the damage caused on these materials by abrasion. Within the context of geogrids, the evaluation of the damage resulting from installation activities or from the exposure to mechanical damage under repeated loading and abrasion tests is usually accomplished by analyzing the variations occurring in their tensile strength [13,16,17,19,20,21,22,24,25]. It is worth mentioning that although these methods (EN ISO 10722 [15] and EN ISO 13427 [26]) have been widely used by several authors to help understand the effect of mechanical damage under repeated loading and abrasion on geosynthetics, the truth is that a clear correlation between the damage caused on the materials by the laboratory tests and the damage occurring on site does not exist. Therefore, it is difficult to extrapolate from the results obtained in those tests what really happens in field conditions.

Although the effect of mechanical damage under repeated loading on the resistance of geosynthetics against abrasion has been analyzed in previous studies [13,14,24,25], there is a lack of investigations dedicated exclusively to addressing this phenomenon in geogrids. This work is a contribution to bridge that gap. In the previously mentioned investigations, it was noticed that abrasion is responsible for producing meaningful damage to geosynthetics, leading to losses in their tensile strength. In some cases, the losses were higher when, prior to the exposure to abrasion tests, the geosynthetics were submitted to mechanical damage under repeated loading tests. This reveals the importance of deepening the knowledge about the effect of mechanical damage under repeated loading on the abrasion process.

The main purpose of this work was to study the resistance of geogrids with different structures (extruded and woven) against abrasion, before and after being exposed to mechanical damage under repeated loading. To accomplish such a goal, besides performing mechanical damage under repeated loading tests in those materials, undamaged samples and damaged samples of geogrids resulting from the single exposure to mechanical damage under repeated loading tests were submitted to abrasion tests. One of the geogrids was chosen to be tested on both sides in order to understand the effect of the side of exposure on the degradation suffered. The damage incurred during the degradation tests was evaluated by visual inspection and by monitoring changes in tensile behaviour of the geogrids. Furthermore, reduction factors were determined based on the changes found in the tensile strength of the geogrids.

The research included in this work, which was focused on geogrids, followed the study developed by Almeida et al. [13] in which different geosynthetics were used to look into the effect of mechanical damage under repeated loading on the resistance against abrasion. The following section will provide the aspects associated with the setup of the experimental program used to fulfil the aims of this work, namely: (1) evaluation of the isolated and combined effects of mechanical damage under repeated loading and abrasion on the degradation suffered by the geogrids; (2) assessment of the influence of mechanical damage under repeated loading in the process of abrasion of the geogrids; and (3) evaluation of the adequacy of different methods to determine the reduction factors accounting for the combined effect of mechanical damage under repeated loading and abrasion.

## 2. Experimental Program

**Figure 1:**
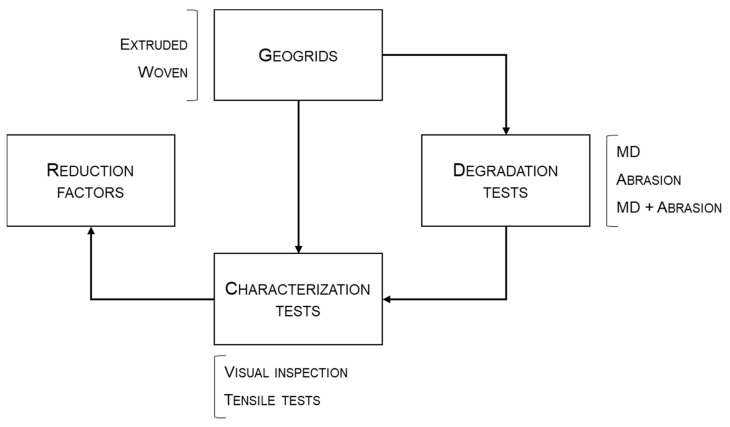
Structure of the experimental program. (MD—mechanical damage under repeated loading).

The experimental program (Figure 1) of this investigation consisted of submitting three geogrids with different properties to mechanical damage (MD) under repeated loading tests (hereinafter MD tests) and to abrasion tests in accordance with EN ISO 10722 [15] and EN ISO 13427 [26], respectively. Initially, the geogrids were exposed in isolation to the aforementioned degradation tests, followed by the successive exposure to MD and abrasion tests (according to this order). For each geogrid, the experimental program involved the use of twenty specimens, in accordance with the following plan: (a) five undamaged specimens; (b) five for the single exposure to MD tests; (c) five for the single exposure to abrasion tests; and (d) five for the successive exposure to both degradation tests. It is important to highlight that one of the geogrids was tested on both sides. Therefore, in this case, thirty-five specimens were tested (the five undamaged specimens were used as a reference for the different sides tested). The sampling and preparation of test specimens followed the guidelines of EN ISO 9862 [27].

The evaluation of the damage suffered by the geogrids after being submitted to the degradation tests was based on two characterization tests. Initially, a visual inspection of the tested specimens targeting the detection of visible damage in their structure was carried out, which was useful to provide an insight regarding the possible existence of changes in the properties of the geogrids. Afterwards, tensile tests were performed to evaluate the short-term tensile behaviour of the materials. The comparison between the results obtained for the undamaged and damaged samples was a tool to assess the level of degradation of the geogrids and to validate the conclusions drawn during the visual inspection.

The work plan also included the determination of reduction factors accounting for the isolated and combined effect of MD and abrasion. The reduction factors for the combined action of the two degradation agents were determined in accordance with two different approaches. The first one consisted of directly obtaining the reduction factors from the successive exposure to both degradation agents. In the second approach, the reduction factors were determined through the multiplication of the reduction factors obtained in isolation for each degradation agent.

### 2.1. Geogrids

**Figure 2:**
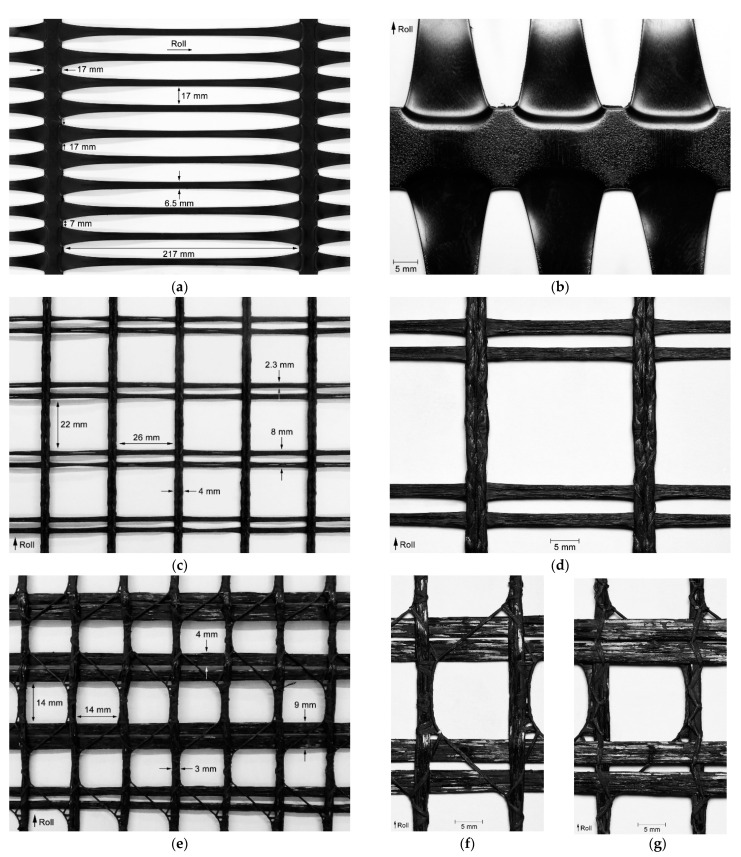
Geogrids: (**a**) overview of extruded geogrid (EG); (**b**) close view of EG; (**c**) overview of woven geogrid I (WG-I); (**d**) close view of WG-I; (**e**) overview of woven geogrid II (WG-II); (**f**) close view of side A of WG-II; (**g**) close view of side B of WG-II. (Side A—side with the machine direction ribs standing out; Side B—side with the cross-machine direction ribs standing out).

The experiments carried out in this work involved the use of a uniaxial extruded geogrid (EG) and two biaxial woven geogrids (WG-I and WG-II), which were supplied in roll form. The raw materials, the tensile strengths (T), and the elongations at maximum load (E_ML_) of the geogrids are displayed in Table 1 (these tensile properties were determined in accordance with EN ISO 10319 [28] in the machine direction of production of the geogrids). In addition, approximate measures of the width of the ribs in the machine and cross-machine directions of production of the geogrids (hereinafter designated by machine and cross-machine direction ribs, respectively), as well as aperture sizes and the indication of the machine directions of production (by means of roll arrows), are provided in Figure 2. The ribs of the woven geogrids had a polymeric coating. Because of its manufacturing process, EG had no coating.

WG-II was the geogrid with the lowest tensile strength, and for that reason it was chosen to be tested on both sides, which were designated by sides A (Figure 2f) and B (Figure 2g). The side A of WG-II corresponded, in structural terms, to the tested side of WG-I. The manufacturing process of woven geogrids leads to a structure in which the machine direction ribs stand out on one side (designated as side A in this work), whereas the cross-machine direction ribs are displayed in the opposite side. Therefore, there is the possibility of the woven geogrids manifesting different behaviours depending on the exposure side to the degradation tests. Regarding EG, since no differences existed between the sides of its structure, the previously mentioned study addressing different sides was not relevant.

The manufacturers of these type of geogrids recommend their use in different contexts. EG can be employed for high strength soil reinforcement in wall and slope applications. WG-I is a soil reinforcement material to be used, for example, in base and sub-base courses of roadway and railway infrastructures, geosynthetic-reinforced soil structures, or embankments on piles. Finally, WG-II is applied in the stabilization of base and sub-base courses of different types of pavements, namely: (1) permanent traffic areas (road pavements, parking areas, industrial facilities); (2) temporary roads (access roads, service roads); (3) working platforms (land sites for construction equipment, infrastructure landfills for buildings); and (4) railways (rehabilitation of existing lines, implementation of new lines). It is important to highlight that the activities carried out during the installation process of the geogrids in the previously mentioned applications can cause damage on their structure. In addition, the materials can also be submitted to abrasive actions over time.

### 2.2. Degradation and Characterization Tests

#### 2.2.1. Mechanical Damage under Repeated Loading Tests

**Figure 3:**
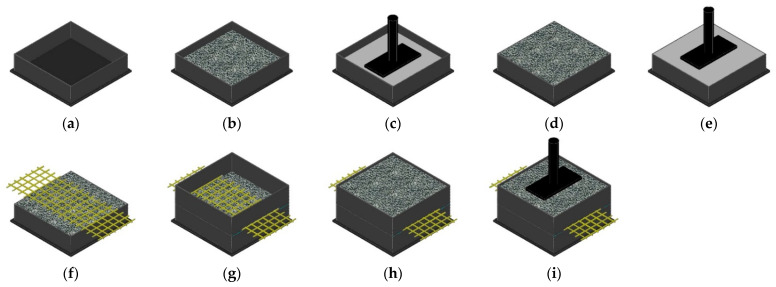
MD test assembly sequence: (**a**) lower box; (**b**) placement and (**c**) compaction of the first sublayer of *corundum*; (**d**) placement and (**e**) compaction of the second sublayer of *corundum*; (**f**) placement of the specimen (in yellow); (**g**) upper box assembly; (**h**) placement of the loose layer of *corundum*; (**i**) application of the dynamic loading.

A laboratory prototype developed at the Faculty of Engineering of the University of Porto (FEUP), Porto, Portugal was employed to perform the MD tests. The equipment included the following elements: (1) two boxes (lower and upper) with a square base of 300 mm and a height of 87.5 mm, in which the standard aggregate (*corundum*) was placed; (2) a metal loading plate with a length and a width of 200 and 100 mm, respectively, for applying the loads; (3) a hydraulic power pack to provide the oil to the load frame targeting the application of loads; and (4) an electronic system for controlling the loads. The experimental procedure of the MD tests is shown in Figure 3.

The MD tests started by the placement of two layers of *corundum* (aggregate made from aluminium oxide with a particle size distribution ranging from 5 to 10 mm) with a height of 37.5 mm in the lower box (Figure 3b,d). These two layers were introduced separately into the lower box (Figure 3a) and each one of them was submitted to a compaction process (Figure 3c,e) that involved applying a load of 200 ± 2 kPa for 60 s over a metal square plate with a side of 295 mm. The next step was the positioning of the specimen of the geogrid (Figure 3f), followed by the placement of the upper box (Figure 3g). The upper box was then filled with a loose layer of *corundum* with a height of 75 mm (Figure 3h), and a vertical cyclic loading between 5.0 ± 0.5 kPa and 500 ± 10 kPa was applied, by means of the metal loading plate, at a frequency of 1 Hz for 200 cycles (the loading conditions were in accordance with EN ISO 10722 [15]) (Figure 3i). At the end of the test, the upper box was emptied and removed in order to collect the specimen of the geogrid without causing further damage. Within the context of the MD tests performed on woven geogrids, it is mentioned throughout the text that sides A or B were tested. These references are used to make clear which side was faced up to the loose layer of *corundum* during these tests.

#### 2.2.2. Abrasion Tests

**Figure 4:**
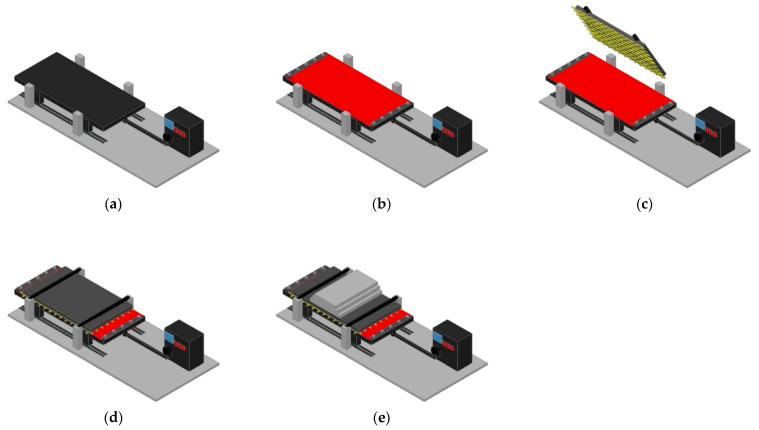
Abrasion test assembly sequence: (**a**) sliding table; (**b**) attachment of the sheet of abrasive (in red); (**c**) geogrid specimen (in yellow) installed in the stationary platform; (**d**) placement of the stationary platform and (**e**) placement of the metal weight (grey plates).

The abrasion tests were also conducted on a laboratory prototype developed at FEUP, Porto, Portugal. The equipment consisted of a sliding table connected to a drive shaft (Figure 4a), a stationary platform, and a metal weight (which was used to apply a pressure over the specimen of the geogrid). The first step of the test was the attachment of a sheet of a P100 abrasive to the sliding table (Figure 4b). Afterwards, the stationary platform, in which the specimen of geogrid was previously installed, was placed above the sliding table (Figure 4c,d), and the metal weight was positioned over the stationary platform (Figure 4e). Finally, the abrasion process was triggered, and the sliding table initiated a cyclic uniaxial movement along a horizontal axis controlled by the drive shaft, under a pressure of 6 kPa for 750 cycles. It is important to highlight that each cycle consisted of a double passage of the abrasive through the specimen of geogrid, i.e., a back-and-forth linear motion.

#### 2.2.3. Tensile Tests

**Figure 5:**
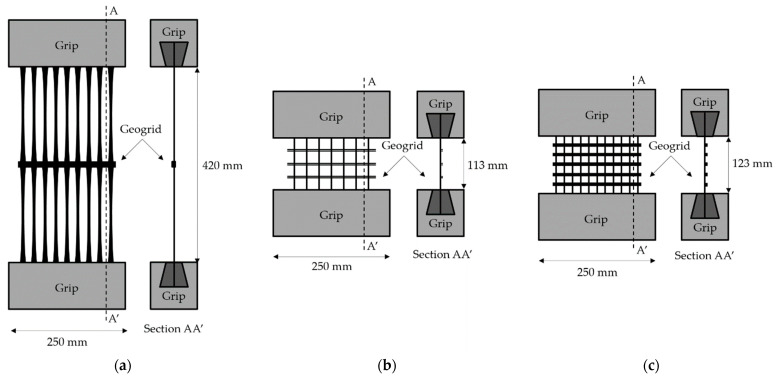
Setup of the tensile tests carried out on geogrids: (**a**) EG; (**b**) WG-I; and (**c**) WG-II.

The tensile tests were carried out in accordance with EN ISO 10319 [28] in the machine direction of production of the geogrids on a LR50K testing machine from Lloyd Instruments (Bognor Regis, UK) fitted with a load cell of 50 kN (accuracy of ±0.5% determined in accordance with ISO 7500-1 [29]), at a displacement rate of 20 mm·min^−1^. A draft of the setup of the specimens of the different geogrids and the grips used in the tensile tests is provided in Figure 5. The dimensions of the specimens were defined taking into account the recommendations of EN ISO 10319 [28], which indicates the use of specimens with a width of, at least, 200 mm. The geogrids were tested with the following number of ribs in the machine direction of production: (a) EG with 9 ribs (43 ribs per meter) (Figure 5a); (b) WG-I with 7 ribs (34 ribs per meter) (Figure 5b); and (c) WG-II with 11 ribs (59 ribs per meter) (Figure 5c). The specimens were attached to the testing machine with serrated wedge grips with screw clamping. The outcomes of these tests were used to figure out the tensile strength (T, in kN·m^−1^) and the elongation at maximum load (E_ML_, in %) of the geogrids.

### 2.3. Data Processing

The values of the two parameters found in the tensile tests (T and E_ML_) resulted from the arithmetic mean of each set of five specimens of geogrids in accordance with the experimental program previously described in Section 2. The results are presented with 95% confidence intervals in accordance with Montgomery & Runger [30]. In addition, the variation of the tensile strength (ΔT, in %) after the degradation tests was determined in accordance with Equation (2):

(2) $$\Delta T=\frac{T_{\left(\mathrm{Damaged}\right)}}{T_{\left(\mathrm{Undamaged}\right)}}\times 100-100,$$

where T_(Damaged)_ is the tensile strength of the geogrids after being exposed to the degradation tests, whereas T_(Undamaged)_ is its counterpart resulting from the tensile tests performed in undamaged specimens.

Reduction factors (RFs) were calculated from Equation (3) considering the isolated and combined effect of the degradation agents on the tensile strength of the geogrids.

(3) $$\mathrm{RF}=\frac{T_{\left(\mathrm{Undamaged}\right)}}{T_{\left(\mathrm{Damaged}\right)}},$$

## 3. Results and Discussion

### 3.1. Visual Inspection

The types of damage caused by the degradation tests on the geogrids were the removal of parts of the polymeric coating (in woven geogrids), cuts in fibres, abrasion, punctures, the tearing of ribs, and the breaking of junctions. The occurrence and level of severity of these forms of damage depended on the degradation test the geogrids were exposed to, on their structure (extruded or woven), and on the tested side. Table 2 displays information about the types of damage, and their respective intensity, observed in the geogrids after the degradation tests (three levels of intensity were defined through the following symbols: “+”—low; “++”—medium; and “+++”—high). The symbol “—” was used in the case of a certain type of damage not being detected.

**Figure 6:**
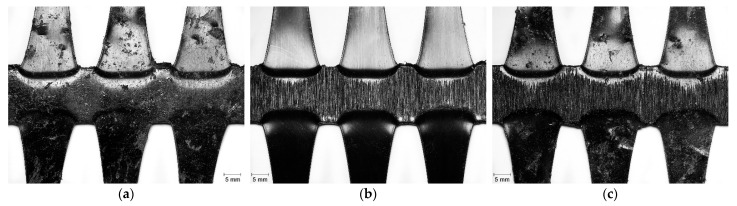
Visual inspection of EG after the degradation tests: (**a**) single exposure to MD tests; (**b**) single exposure to abrasion tests; (**c**) successive exposure to MD and abrasion tests.

EG had considerably higher resistance to damage than the woven geogrids, as can be observed in Table 2. After the MD tests, only some punctures in the ribs of EG were observed (Figure 6a). The punctures were caused by the sharp edges of the particles of the standard aggregate used in the MD tests (*corundum* is an aggregate with uniform, angular, rough, and hard particles). The abrasion tests also caused relatively minor damage (Figure 6b). Indeed, the machine direction ribs were not affected by these tests. On the other hand, the cross-machine direction ribs and the junctions of EG became worn out (only these elements came into contact with the P100 abrasive since they were thicker: the cross-machine direction ribs were roughly 2.5 times thicker than the machine direction ribs). It should also be mentioned that the abrasive cycles caused a slight reduction in the thickness of the cross-machine direction ribs. The successive exposure to both degradation tests led to the occurrence of the combination of the two types of damage observed after each individual test, i.e., punctures in the ribs and abrasion in the cross-machine direction ribs and in the junctions (Figure 6c).

**Figure 7:**
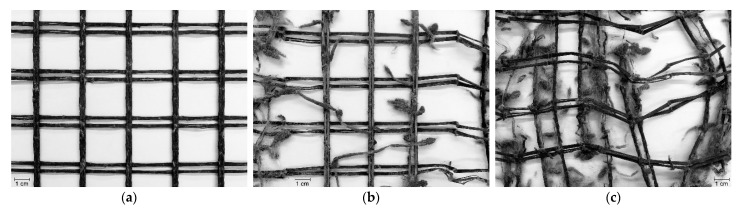
Visual inspection of WG-I after the degradation tests: (**a**) single exposure to MD tests; (**b**) single exposure to abrasion tests; (**c**) successive exposure to MD and abrasion tests.

**Figure 8:**
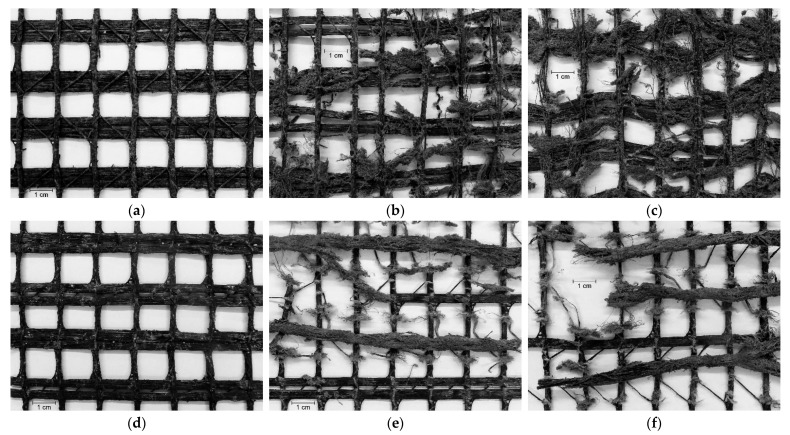
Visual inspection of WG-II after the degradation tests: (**a**) single exposure to MD tests (side A); (**b**) single exposure to abrasion tests (side A); (**c**) successive exposure to MD and abrasion tests (side A); (**d**) single exposure to MD tests (side B); (**e**) single exposure to abrasion tests (side B); (**f**) successive exposure to MD and abrasion tests (side B).

The effect of the degradation tests on WG-I and WG-II was much more severe compared to EG (Table 2). During the MD tests, both woven geogrids suffered some cuts in fibres, punctures in the ribs, and the removal of parts of the polymeric coating (Figure 7a and Figure 8a,d). These types of damage resulted from the action of the particles of *corundum* (the reasons for such an outcome were provided in the previous discussion for EG). The abrasion tests had a much more pronounced effect on the structure of WG-I and WG-II than the MD tests. The abrasion induced to the geogrids during those tests led to the removal of parts of the ribs’ polymeric coating, the generation of a large amount of cuts in fibres, the tearing of many ribs, and the rupture of junctions (Figure 7b and Figure 8b,e). Besides that, the abrasion tests also caused a decrease in the thickness of the ribs (the fibres forming the ribs were broken and gradually removed throughout the abrasive cycles). The successive exposure to both degradation tests caused the same types of damage observed after the single exposure to abrasion tests and also some punctures as observed after the single exposure to MD tests. However, the damage caused by the abrasion tests seemed to have been slightly enhanced by the damage initially imposed during the MD tests (Figure 7c and Figure 8c,f).

Comparing the woven geogrids tested on the same exposure side (i.e., the side with the machine direction ribs directly exposed to the degradation agents), the relevant feature was that the damage induced to WG-II (side A) during the abrasion tests was slightly more meaningful than in WG-I (the difference was the existence of a lower level of damage in terms of tearing and breaking of junctions in WG-I). The slightly higher degradation found in WG-II (side A) may be ascribed to the smallest width of the machine direction ribs (4 mm in WG-I and 3 mm in WG-II) or to the type of raw material used for manufacturing the materials (polyester in WG-I and polypropylene in WG-II). If different, the thickness of the machine direction ribs could also have been responsible for differences in the damage suffered by the geogrids during the abrasion tests. However, these elements had approximately the same thickness in WG-I and WG-II. No relevant differences were found in the damage suffered by the woven geogrids in the case of the single exposure to MD tests. The same outcome was noticed after the successive exposure to MD and abrasion tests.

The side of the geogrids directly exposed to the damaging actions is an aspect that can influence the damage suffered by the materials during the degradation tests. For geogrids with no structural differences between sides, as EG, the same resistance to damage regardless of the side of exposure is expected. However, for woven geogrids with sides with different characteristics, the side of exposure is an issue that might influence the level of damage suffered by the materials. The manufacturing process of woven geogrids often leads to a structure in which the machine direction and cross-machine direction ribs are overlapped and stitched with yarns (this process results in a structure where the machine direction ribs stand out on one side). This was the case with both woven geogrids studied in this work. To analyse this issue, the single and successive exposures to the degradation tests were conducted on side B of the WG-II (the opposite side to the one in which the machine direction ribs stand out). As mentioned in Section 2.1, the reason for using WG-II in this analysis was its lower tensile strength compared to WG-I.

The types of damage observed on both sides of the WG-II were similar. Considering the exposure to MD tests, no differences were noticed between sides A and B. This was an expected outcome since the geogrids were exposed on both sides simultaneously during the MD tests (the specimens were entirely surrounded by particles of *corundum*). However, a different outcome was found after the abrasion tests. The most relevant feature was the fact that the machine direction ribs of WG-II suffered more damage when side A was tested. Indeed, it was noticed that the tearing of machine direction ribs was more significant compared to side B. The very same conclusion can be drawn with regard to the successive exposure to both degradation tests. This occurred because, during the abrasion tests on side A, the machine direction ribs were directly exposed to the damaging actions (Figure 2f). On the contrary, when side B of WG-II was exposed to those tests, the cross-machine direction ribs conferred protection to the machine direction ribs against the damaging actions (Figure 2g) (this circumstance led to a higher level of damage suffered by these ribs). It is important to mention that despite the existence of some visible differences, the abrasion tests and the successive exposure to MD tests and abrasion tests resulted in a significant degradation of the cross-machine direction ribs of WG-II when both sides A and B were tested.

### 3.2. Tensile Behaviour

The tensile properties of the geogrids after being submitted to the single and successive exposures to the degradation tests can be found in Table 3. The results revealed the existence of relevant changes in the tensile behaviour of the materials. It is worth remembering that since the geogrids were tested in the machine direction of production, the elements responsible for the tensile strength were the machine direction ribs.

As expected, considering the damage detected in the visual inspection (only a few punctures, and abrasion in the cross-machine direction ribs and in the junctions), the single and successive exposures to MD and abrasion tests caused only relatively minor changes in the tensile properties of EG. In the case of the single exposure to MD tests, a ΔT of −11.5% was found in the tensile strength compared to the undamaged sample. Simultaneously, elongation at maximum load changed from 13.7 to 12.2%. Concerning the single exposure to abrasion tests, and taking into account the 95% confidence intervals, it was not possible to conclude if the slight variation in the tensile properties of EG resulted from the damage suffered (abrasion in the cross-machine direction ribs and in the junctions) or if it might be associated with the existence of some heterogeneity in the material. The tensile properties obtained after the successive exposure to both degradation tests were similar to those observed after the single exposure to MD tests, which supports the idea of the non-occurrence of relevant damage during the abrasion tests. The understanding was that the changes observed in the tensile properties of EG after the single exposure to MD tests and after the successive exposure to both degradation tests were caused, almost exclusively, by the damage imposed during the MD tests. Indeed, the variation observed in tensile strength after the single exposure to MD tests and after the successive exposure to both degradation tests were similar (ΔT of −11.5 and −11.8%, respectively). The changes found in elongation at maximum load were also identical.

WG-I presented good resistance against the single exposure to MD tests. Indeed, the cuts in fibres, the punctures, and the removal of parts of the polymeric coating caused by the MD tests did not lead to significant changes in the tensile properties of WG-I. On the contrary, the tensile behaviour of WG-I was severely affected after the single exposure to abrasion tests (ΔT of −80.9% and a reduction in elongation at maximum load from 12.1 to 9.5%). The scale of these changes was in agreement with the damage observed during visual inspection, namely, the loss of parts of the polymeric coating, the considerable number of cuts in fibres, the tearing of ribs, and the rupture of junctions. The successive exposure to both degradation tests caused an even higher reduction in the tensile strength (ΔT of −95.3%). Regarding elongation at maximum load, no relevant changes were found compared to the single exposure to abrasion tests. Although the single exposure to MD tests did not foster relevant changes in the tensile properties of WG-I, it is likely that the few cuts in fibres, the punctures, and also the slight loss of the polymeric coating might have promoted the occurrence of more significant damage during the following abrasion tests, which resulted in a further strength loss (compared to the single exposure to abrasion tests). Indeed, the damage caused by the MD tests might have left the geogrid more susceptible to degradation during the following abrasion tests. Therefore, the effect (intensity of damage and deterioration of tensile behaviour) of the successive exposure was more pronounced than the sum of the isolated effects of each degradation test.

The tensile behaviour of WG-II was also affected after the single exposures to MD and abrasion tests. The losses caused by the MD tests in the tensile strength of WG-II, when different sides were tested, were similar (ΔT of −21.0 and −22.4% for sides A and B, respectively). The decreases observed in elongation at maximum load were also identical, independently of the tested side. This was an expected outcome taking into account that both sides of the geogrids were exposed concurrently to the action of the particles of *corundum*, independently of the side of the geogrid facing up in the MD tests. This behaviour also corroborates the relatively low level of degradation observed during the visual inspection, namely, minor cuts in fibres, punctures, and a slight removal of the polymeric coating. A different outcome was found after the single exposure to abrasion tests. WG-II was severely affected when side A was exposed directly to the abrasive, resulting in a ΔT of −91.2%, which was considerably more significant compared to the results for side B, in which a ΔT of −59.6% was found. Similar to tensile strength, elongation at maximum load was also affected more on side A. This was not a surprise given the occurrence of a higher level of tearing of the ribs when side A was tested (Table 2). The changes in the tensile behaviour were even more relevant in the case of the successive exposure to both degradation tests. Similar to what happened for WG-I, the damage induced to WG-II during the MD tests might have contributed to increase its degradation throughout the following abrasion tests (the samples forwarded to abrasion tests after being submitted to MD tests were more susceptible to degradation), and, as consequence, higher losses occurred in tensile strength (ΔT of −96.9 and −79.2%, for sides A and B, respectively).

Within the context of testing different sides of WG-II, the conclusion was that the side of exposure had a relevant influence on the damage (i.e., deterioration of tensile properties) induced by the abrasion tests. The results indicate that, in the case of side A, in which the machine direction ribs stood out on the side of exposure to the abrasive cycles, more pronounced changes occurred in the tensile behaviour of WG-II. On the other hand, when the opposite side was tested, the level of degradation suffered by the machine direction ribs was not so accented. These elements, which were responsible for providing the tensile strength since WG-II was tested in the machine direction of production, were protected from direct contact with the abrasive because of the cross-machine direction ribs displayed over them. Furthermore, it should be mentioned that the outcomes could be different in the case of WG-II being tested in the cross-machine direction of production. It is likely that the damage noticed for side A would be lower compared to side B, since the mechanism of protection of the ribs would be the opposite of the case studied in this work.

Besides the effect of the side of exposure, the results also showed that the degrees of change in the tensile properties differed according to the type of geogrid. EG presented higher resistance against damage than the woven geogrids. This outcome resulted from its manufacturing process and geometry. Extruded geogrids are formed by a continuous polymeric structure that make them hard and stiff, whereas woven geogrids are composed by bundles of agglomerated fibres making them softer and more flexible compared to extruded geogrids. By themselves, the different manufacturing processes (which lead to different structures) can justify the differences observed in the behaviour of the geogrids when submitted to the MD and abrasion tests. However, the higher resistance against damage of EG (especially when exposed to abrasive actions) can also be related to its geometry. Indeed, the ribs of EG had different thicknesses (the cross-machine direction ribs were thicker than their counterparts displayed through the machine direction of production). Because of that, during the abrasion tests, only the cross-machine direction ribs suffered degradation (the machine direction ribs were not in contact with the P100 abrasive). Moreover, the reduction in thickness of these ribs did not result in relevant changes in the tensile properties of EG since the resistant elements were the machine direction ribs, which remained undamaged. As a consequence, the abrasion tests (performed in accordance with EN ISO 13427 [26]) may not be a good approximation of the real mechanism occurring on-site since the effective area of EG submitted to abrasive actions was limited by the testing method. The larger contact area between the surfaces of the woven geogrids and the P100 abrasive might also have contributed to the greater damage suffered by these materials when compared to EG. Considering what it is expected to happen during service life, it is important to mention that the abrasion tests might have an exaggerated damaging effect on the woven geogrids. However, there are no studies establishing correlations between the outcomes of the tests based on EN ISO 13427 [26] and on-site conditions. Therefore, special attention should be given to the circumstance in which woven geogrids expected to be exposed to abrasive actions are applied in engineering applications.

The woven geogrids exhibited a similar behaviour when exposed to the degradation tests in the sense that both were not meaningfully affected when exposed to MD tests but suffered significant damage after being submitted to abrasion tests. The successive exposure to the two degradation tests resulted in a further increase in degradation. However, a trend for a higher degradation (i.e., higher deterioration of the tensile behaviour) of WG-II compared to WG-I was noticed, specifically after the single exposures to MD tests and abrasion tests. Two features can be highlighted to try to explain the different, although not very pronounced, resistances against damage presented by the woven geogrids within this context. The first one is that the machine direction ribs of WG-II had a lower width compared to WG-I, which might have led to higher difficulties of these elements to resist against the damaging actions. On the other hand, the raw materials used for manufacturing the woven geogrids were different (polyester was employed in WG-I and polypropylene in WG-II), which might also be a reason that contributed to the different degradation of the tensile behaviour of the woven geogrids. However, there is no supporting data to confirm, or discard, the previous hypothesis.

### 3.3. Reduction Factors

The RFs accounting for the isolated and combined effect of MD and abrasion are provided in the current section. It is important to underline that the RFs determined in this work resulted from specific conditions associated with standard tests, which might not correspond to the conditions that the geogrids will be exposed to on-site, especially the conditions of the abrasion tests, as mentioned in the previous section. For that reason, these RFs should not be a reference for the design. The RFs found in this work only served the purpose of comparing the outcomes of two different methods to account for the combined effect of MD and abrasion: one resulting from the successive exposure to the degradation agents and another that is addressed below. Table 4 provides the RFs resulting from the single exposures to the MD (RF_MD_) and abrasion (RF_ABR_) tests, as well as the RFs accounting for the successive exposure to both degradation tests (RF_MD+ABR(SE)_). These RFs were determined in accordance with Equation (3), which relates the tensile strength of the geogrids before and after their exposure to the degradation tests. Because of that, the observations made in Section 3.2 about the tensile strength of the geogrids can be transposed to this discussion.

The RFs obtained for EG after the single and successive exposures to MD and abrasion tests were around 1, which reflects the good resistance of this geogrid against those degradation tests. Furthermore, WG-I and WG-II were more damaged by the degradation tests than EG, thus, higher RFs were obtained for the woven geogrids.

The values of RF_MD+ABR(SE)_ were compared with the RFs resulting from a common method to account for the combined effect of the two degradation agents, which consisted in multiplying the RFs obtained in isolation for the effect of each one of those degradation agents. Equation (4) materializes this method:

(4) $${\mathrm{RF}}_{\mathrm{MD}+\mathrm{ABR}(M)}={\mathrm{RF}}_{\mathrm{MD}}\times {\mathrm{RF}}_{\mathrm{ABR}},$$

where RF_MD+ABR(M)_ is the resulting reduction RF for the combined effect of MD and abrasion. The comparison between the RFs calculated through Equation (4) (RF_MD+ABR(M)_) and those obtained through the successive exposure to both degradation tests (RF_MD+ABR(SE)_), which are displayed in Table 4, allowed to evaluate if the multiplication of the two RFs resulting from the single exposure to each one of them is an appropriate method to account for the respective combined effect.

The RFs obtained with both methods were of the same order for EG. Thus, the multiplication of the RFs considering the isolated effect of MD and abrasion correctly represented the combined effect of the degradation agents. This was an expected outcome taking into account the minor degradation caused by the MD and abrasion tests. On the contrary, the RFs obtained for the woven geogrids after the successive exposure to both degradation agents were higher than those resulting from the multiplication of the RFs obtained in isolation for each agent. In this case, the latter method was not capable of accounting for the damage (i.e., changes in tensile strength) caused by the combined action of the degradation agents, disregarding the interactions (synergisms) occurring between them. For example, the single exposure to MD tests did not lead to significant changes in the tensile strength of WG-I. However, these tests caused some damage in the ribs of the geogrid that contributed to increase the destructive effect of the subsequent abrasion tests. This effect was observed for WG-I and for WG-II, independently of the side of exposure. In the case of WG-I, the RF obtained through the multiplication of RF_MD_ by RF_ABR_ (5.39) was considerably lower than the RF resulting from the successive exposure (21.07). A similar conclusion could be obtained for WG-I and WG-II. This circumstance allows to mention that the use of Equation (4) was not able to represent correctly the combined effect of MD and abrasion.

The excessively high values found for many RFs (e.g., 11.34 and higher) are inadequate and would never be used for the design (RFs around 5 are already considered high). Within this context, it is worth mentioning that geogrids with this on-site behaviour would never be considered suitable to be used under any circumstances, since they would not be able to accomplish the functions for which they were designed (alternative reinforcement materials, including other geogrids, would have to be used). As is understandable, these RFs result from testing conditions that might not represent the reality found on-site. It is important to stress that no attempts were carried out to obtain correlations between the laboratory tests and on-site conditions. The definition of the RFs to be used in the design must be carried out case by case, always taking into account the specificities of each project.

### 3.4. Comparison between the Reduction Factors Obtained in This Work with Findings of Other Investigations

**Figure 9:**
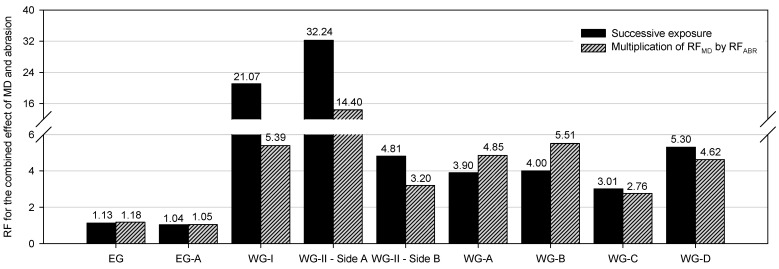
Comparison between reduction factors (RFs) obtained in this work and in other studies [13,24,25] for the combined effect of MD and abrasion on geogrids. (EG-A—polypropylene extruded biaxial geogrid with tensile strength of 46.6 kN·m^−1^; WG-A—polyester woven geogrid with tensile strength of 44.4 kN·m^−1^; WG-B—polyester woven geogrid with tensile strength of 47.5 kN·m^−1^; WG-C—polypropylene woven geogrid with tensile strength of 44.4 kN·m^−1^; WG-D—polyester woven geogrid with tensile strength of 39.8 kN·m^−1^).

To the best of our knowledge, Rosete et al. [24], Pinho-Lopes and Lopes [25], and Almeida et al. [13] are the only studies that addressed the single and combined effect of MD and abrasion in geogrids. Figure 9 provides the RFs determined in those investigations, as well as the RFs obtained in this work, allowing a quick comparison. In all cases, the RFs were obtained based on the changes that occurred in the tensile strength of the geogrids.

The RFs (near 1) obtained by Rosete et al. [24] for the combined effect of MD and abrasion on a polypropylene extruded biaxial geogrid (designated by EG-A in Figure 9) with a tensile strength of 46.6 kN·m^−1^ were similar to those obtained in this work for EG (it is important to highlight that no relevant differences were found between the RFs obtained through the different determination methods). Therefore, in both works, the extruded geogrids, one uniaxial and the other biaxial, had good resistance against the damage induced by these two degradation agents.

The RFs presented by Rosete et al. [24] and Pinho-Lopes and Lopes [25] for two polyester woven geogrids (designated by WG-A and WG-B in Figure 9, with tensile strengths of 44.4 and 47.5 kN·m^−1^, respectively) are not in agreement with the results obtained in this work. In those studies, the RFs obtained through the multiplication of the RFs obtained in isolation for each degradation agent were higher than those resulting from the successive exposure of the geogrids to MD and abrasion. By contrast, the RFs obtained by Almeida et al. [13] for two woven geogrids, identified in Figure 9 as WG-C (made from polypropylene; tensile strength of 44.4 kN·m^−1^) and WG-D (manufactured with polyester; tensile strength of 39.8 kN·m^−1^), are consistent with the outcomes of this work, i.e., the RFs obtained after the successive exposure to both degradation agents were higher compared to the RFs resulting from the multiplication of the RFs accounting for the effects of the single exposures to MD tests and abrasion tests. It is also worth mentioning that the higher RFs found in the different works for the woven geogrids, compared to the extruded ones, reveal their lowest resistance against the damage caused by MD and abrasion.

Taking into account the data displayed in this section, it was noticed that the multiplication of the RFs obtained for the isolated effects of MD and abrasion seemed to be only able to provide a good approximation for the combined effect of the degradation agents when EG and EG-A were tested, which was the case wherein the materials suffered only minor damage. In addition, this method revealed to be conservative for WG-A and WG-B, since its application led to higher RFs compared to their counterparts directly resulting from the successive exposures. By contrast, the previously mentioned method consisting of multiplying the RFs obtained in isolation was not able to account for the changes in tensile strength caused by both degradation agents acting simultaneously in WG-I, WG-II, WG-C, and WG-D. Indeed, in these cases, the RFs obtained directly through the successive exposure were higher. Therefore, considering that the method in which the RFs obtained in isolation are multiplied might not be suitable to accurately represent the combined effect of the degradation agents, it appears reasonable that future analysis within the context of soil reinforcement with geogrids involving the study of the combined effect of different degradation agents takes into account, whenever possible, the outcomes of successive exposures. Results have shown that the process of abrasion of geogrids can be affected by prior exposure to mechanical damage under repeated loading.

## 4. Conclusions

This work studied the damage suffered by three geogrids (one extruded and two woven) after being submitted to single exposures to MD and abrasion tests and to successive exposure to both degradation tests. Upon conclusion of these tests, the geogrids were submitted to a visual inspection in order to identify the types of damage suffered, as well as their level of intensity. The following types of damage were noticed: cuts in fibres, punctures, tearing of ribs, abrasion, the breaking of junctions, and the removal of parts of the polymeric coating. The occurrence and severity of these types of damage depended on the degradation test, on the type of geogrid (extruded or woven), and on the side of the geogrid exposed to the degradation tests.

The tensile tests that were performed on geogrids revealed that the MD tests caused relatively minor changes in their tensile behaviour (only the woven geogrid WG-II suffered some deterioration of its tensile properties). On the contrary, the single exposure to abrasion tests led to substantial decreases in the tensile properties of the woven geogrids but not of the extruded geogrid. The successive exposure to both degradation tests led to even more pronounced reductions in the tensile properties of the woven geogrids (once again, no relevant changes were found in the tensile behaviour of the extruded geogrid). Regarding the side of exposure, an issue that has not been evaluated in previous works, the machine direction ribs of WG-II, which were responsible to supply the strength of the geogrid (the materials were tested in the machine direction of production), proved to have a higher resistance against the abrasion tests and the successive exposure to both degradation tests (i.e., lower deterioration of the tensile behaviour) when the tested side was the one in which the cross-machine direction ribs stand out. No differences were found in the case of the MD tests.

The damage caused by the MD tests tended to have a negative effect on the resistance of the woven geogrids against the subsequent abrasion tests, since the initial exposure to MD tests resulted in materials more susceptible to suffer abrasive degradation. Taking into account these findings, designers should be aware of the need of considering the effect of MD (resulting from the activities carried out during the installation on-site) on the future resistance against abrasion of woven geogrids in applications in which the materials might be exposed to long-term abrasive actions. This is the case for geogrids WG-I and WG-II, since they are recommended by the manufacturers to be used in applications in the domain of roadway and railway infrastructures, in which vehicle traffic is responsible for generating cyclic loads. If the performance of the woven geogrids will be affected by the abrasive actions, there is a risk of failure of the applications in which they are going to be applied. The extruded geogrid had the highest resistance against the laboratory damaging actions, which suggests that this type of geogrid may be a better solution for applications in which abrasive actions are expected to occur over time. However, the extrapolation of the obtained results to field conditions is an issue that deserves attention.

RFs for the combined effect of the degradation agents were calculated, based on the changes that occurred in tensile strength of the geogrids, by two different methods. In the case of the extruded geogrid, the RFs determined by both methods were similar. By contrast, the RFs obtained for the woven geogrids by the common method (consisting of multiplying the RFs obtained in isolation to each one of the degradation agents) were lower than those directly found in the successive exposure to MD and abrasion. Therefore, the common method may not be able to correctly represent the combined effect of the degradation agents, which can result in erroneous designs. Targeting the determination of accurate RFs, the degradation that geogrids will experience over time has to be properly estimated. This is of utmost importance to ensure the correct behaviour of the geogrids in any application, including, or not, those in which abrasive actions are prone to occur.

## Figures and Tables

**Table 1:** Properties of geogrids.

Geogrid	Type	Raw Material	T (kN·m^−1^)	E_ML_ (%)
EG	Extruded	High-density polyethylene	53.88 (±1.44)	13.7 (±0.4)
WG-I	Woven	Polyester	44.25 (±1.44)	12.1 (±0.7)
WG-II	Woven	Polypropylene	30.95 (±1.63)	11.2 (±0.6)

EG—extruded geogrid; WG-I—woven geogrid I; WG-II—woven geogrid II. (95% confidence intervals in brackets).

**Table 2:** Damage observed in the geogrids after the degradation tests.

Geogrid	Degradation Test	Types of Damage					
		Cuts in Fibres	Punctures	Tearing	Abrasion	Breaking of Junctions	Coating Removal
EG	MD	N/A ^1^	+	—	—	—	N/A ^1^
	Abrasion	N/A ^1^	—	—	+	—	N/A ^1^
	MD + abrasion	N/A ^1^	+	—	+	—	N/A ^1^
WG-I	MD	+	+	—	—	—	+
	Abrasion	+++	—	++	+++	++	+++
	MD + abrasion	+++	+	+++	+++	+++	+++
WG-II–Side A	MD	+	+	—	—	—	+
	Abrasion	+++	—	+++	+++	+++	+++
	MD + abrasion	+++	+	+++	+++	+++	+++
WG-II–Side B	MD	+	+	—	—	—	+
	Abrasion	+++	—	++	+++	+++	+++
	MD + abrasion	+++	+	++	+++	+++	+++

^1^ N/A—not applicable.

**Table 3:** Tensile properties and variations of the tensile strength of the geogrids after the degradation tests.

Geogrid	Degradation Test	T (kN·m^−1^)	E_ML_ (%)	ΔT (%)
EG	MD	47.66 (±1.04)	12.2 (±0.1)	−11.5
	Abrasion	51.64 (±3.12)	13.0 (±1.0)	−4.2
	MD + abrasion	47.53 (±2.09)	12.4 (±0.4)	−11.8
WG-I	MD	43.06 (±1.39)	12.7 (±0.5)	−2.7
	Abrasion	8.46 (±3.79)	9.5 (±0.7)	−80.9
	MD + abrasion	2.10 (±1.13)	8.6 (±2.4)	−95.3
WG-II–Side A	MD	24.46 (±1.64)	8.6 (±0.5)	−21.0
	Abrasion	2.73 (±0.47)	4.6 (±1.6)	−91.2
	MD + abrasion	0.96 (±0.20)	5.3 (±2.1)	−96.9
WG-II–Side B	MD	24.01 (±1.85)	8.7 (±0.9)	−22.4
	Abrasion	12.50 (±4.12)	6.7 (±1.6)	−59.6
	MD + abrasion	6.44 (±4.35)	6.6 (±0.9)	−79.2

(95% confidence intervals in brackets).

**Table 4:** Reduction factors for the tensile strength of the geogrids.

Reduction Factor	EG	WG-I	WG-II–Side A	WG-II–Side B
RF_MD_	1.13	1.03	1.27	1.29
RF_ABR_	1.04	5.23	11.34	2.48
RF_MD+ABR(SE)_	1.13	21.07	32.24	4.81
RF_MD+ABR(M)_	1.18	5.39	14.40	3.20

## Data Availability

The data presented in this study are available on request from the corresponding author.
